# Support vector machine classification of arterial volume‐weighted arterial spin tagging images

**DOI:** 10.1002/brb3.549

**Published:** 2016-10-07

**Authors:** Yash S. Shah, Luis Hernandez‐Garcia, Hesamoddin Jahanian, Scott J. Peltier

**Affiliations:** ^1^ Functional MRI LaboratoryBiomedical EngineeringUniversity of MichiganAnn ArborMIUSA; ^2^ Department of RadiologyStanford UniversityStanfordCAUSA

**Keywords:** arterial cerebral blood volume, arterial spin labeling, machine learning, support vector machines

## Abstract

**Introduction:**

In recent years, machine‐learning techniques have gained growing popularity in medical image analysis. Temporal brain‐state classification is one of the major applications of machine‐learning techniques in functional magnetic resonance imaging (fMRI) brain data. This article explores the use of support vector machine (SVM) classification technique with motor‐visual activation paradigm to perform brain‐state classification into activation and rest with an emphasis on different acquisition techniques.

**Methods:**

Images were acquired using a recently developed variant of traditional pseudocontinuous arterial spin labeling technique called arterial volume‐weighted arterial spin tagging (AVAST). The classification scheme is also performed on images acquired using blood oxygenation–level dependent (BOLD) and traditional perfusion‐weighted arterial spin labeling (ASL) techniques for comparison.

**Results:**

The AVAST technique outperforms traditional pseudocontinuous ASL, achieving classification accuracy comparable to that of BOLD contrast images.

**Conclusion:**

This study demonstrates that AVAST has superior signal‐to‐noise ratio and improved temporal resolution as compared with traditional perfusion‐weighted ASL and reduced sensitivity to scanner drift as compared with BOLD. Owing to these characteristics, AVAST lends itself as an ideal choice for dynamic fMRI and real‐time neurofeedback experiments with sustained activation periods.

## 1 Introduction

Functional magnetic resonance imaging (fMRI) is a noninvasive technique used for visualization of regional brain activity. Blood oxygenation–level dependent (BOLD) contrast‐based techniques are the most commonly used methods for acquiring fMRI images. The hemodynamic response to neuronal activation entails a temporary increase in blood volume and oxygenation level in the blood. BOLD techniques take advantage of the difference in magnetic properties of oxygenated and deoxygenated blood to generate images and are widely used as a marker for providing reliable information about neural activation (Detre & Wang, 2002). The intensity of obtained images is relative and not individually quantitative because BOLD does not involve direct measurement of any physiological parameter, unless a number of additional measures are collected (Davis, Kwong, Weisskoff, & Rosen, 1998; Hoge et al., 1999). The BOLD signal is sensitive to field inhomogeneities caused by the differences in magnetic susceptibility of air and tissue, which may result in local tissue distortions and signal losses (Sutton, Noll, & Fessler, 2003; Weiskopf, Hutton, Josephs, & Deichmann, 2006). The within‐session slow scanner drift which has been observed in most studies using BOLD imaging (Smith et al., 1999) makes it impractical to use for studies involving neural processes with long activation periods.

The observed signal in arterial spin labeling (ASL) method depends on cerebral blood flow (CBF) alone and is largely independent of the oxygenation level. ASL techniques are less sensitive to local susceptibility artifacts due to the use of shorter echo times (TEs) and show reduced sensitivity to the MR scanner drift since they are subtraction techniques. Unlike BOLD imaging, they are also capable of absolute quantification of CBF in well‐characterized physiological units, but ASL suffers from inadequacies such as low signal‐to‐noise ratio (SNR) and poor temporal resolution.

Since cerebral perfusion is regulated at the arteriolar level, measuring the arterial cerebral blood volume (aCBV) provides useful information about neuronal activation (Lee, Duong, Yang, Iadecola, & Kim, 2001; Mumford & Nichols, 2009). Arterial volume‐weighted arterial spin tagging (AVAST) is a variant of pseudocontinuous arterial spin labeling acquisition (PCASL) technique, which measures aCBV (Jahanian, Peltier, Noll, & Hernandez‐Garcia, 2015). fMRI using physiological parameters such as CBF or CBV, unlike BOLD fMRI, provides a quantifiable contrast and is more closely related to neural activity (Duong et al., 2002; Jin & Kim, 2008; Kim & Kim, 2010).

The short relaxation time of arterial blood causes the tag to decay rapidly resulting in lower SNR while using traditional perfusion‐weighted ASL techniques. AVAST demonstrates superior SNR since the images are acquired while tag is still in the arteries before perfusion. AVAST is based on optimizing the timing parameters of a PCASL sequence such that the subtracted ASL signal is predominantly from the arteries, rather than from the capillaries and the tissue parenchyma. The technique tailors the tagging duration and repetition time (TR) for each subject to achieve a contrast that depends on aCBV with little contribution from tissue perfusion signal by taking advantage of the kinetics of the tag through that subject's vasculature. In other words, the strategy behind AVAST is to design the timing parameters of the PCASL sequence such that the signal is acquired when the tagged spins are primarily still in the arteries before they have filled the capillaries. Hence, there are no delays between the tagging period and the acquisition. The tagging duration (and consequently, TR) is adjusted in a calibration scan until the contribution from tagged spins in the capillary and tissue compartments is the same in both the control and tagged images and therefore can be subtracted out. AVAST exhibits activation detection sensitivity and temporal resolution that is on par with BOLD imaging and an improvement over the standard CBF ASL technique, while preserving its quantitative nature and statistical advantages (Jahanian et al., 2015).

Traditionally, fMRI analysis is generally performed using a univariate approach called statistical parametric mapping or SPM (Friston et al., 1995), which examines the differences in brain activity in a general linear model (GLM) framework. In SPM, each voxel is analyzed using a univariate statistical parametric test and the resulting statistics are assembled into an image that is interpreted as a spatially extended statistical process. Several other whole‐brain mass univariate analysis techniques have been implemented in the past to detect patterns in the brain imaging data (Luo & Nichols, 2003; Smith, 2004). However, in a univariate approach, each voxel's time series is treated independently and it does not take into account inter‐regional correlations, which may be vital in studies of neural systems associated with particular brain function. Also, they do not offer the prospect of employing a predictive learning approach, wherein, a model computed using some training data is then used to predict the outcome for an unseen test example. This approach may be of significant diagnostic relevance.

Contrarily, multivariate pattern analysis (MVPA) techniques process all the data together and make more use of the spatial relationships within the data. They involve the application of sophisticated algorithms to complex patterns generated by the very large number of features that is voxel intensities (Formissano, De Martino, & Valente, 2008). By taking into account the full spatial pattern of brain activity, measured simultaneously at many locations, these methods allow detecting subtle localized effects that may remain unidentified with conventional univariate statistical methods.

Machine learning has been used increasingly to analyze fMRI images, and it involves the use of an algorithm to facilitate learning from examples. In supervised machine‐learning algorithms, there is first a training phase, during which labeled input training samples are used to build a model that captures the relationship between the training samples and the corresponding labels (Pereira, Mitchell, & Botvinick, 2009). This model is then used during the testing phase to compute an output label for any new testing data sample. In typical fMRI applications, machine‐learning algorithms are used to learn a relationship between brain volumes and labels. This learned functional relationship is then used to predict the unseen labels for a new test dataset. Thus, they facilitate a classifier‐based predictive learning framework. Such a setup has been used with fMRI data to enable brain‐state classification (Cortes & Vapnik, 1995; LaConte, Strother, Cherkassky, Anderson, & Hu, 2005; Mitchell et al., 2004).

A number of different machine‐learning techniques have been used for MVPA in functional MRI studies to investigate different neural processes. Representative studies have been summarized below. Support vector machines (SVMs) and linear discriminant analysis (LDA) were applied to successfully classify patterns of fMRI activation observed due to the visual presentation of pictorial cues of various categories of objects (Cox & Savoy, 2003). In another study, several different classifier training methods including Gaussian Naïve Bayes, SVM, and k‐nearest neighbor were explored to learn to decode cognitive states from brain images (Mitchell et al., 2004). Nonlinear SVM and Fisher's LDA have been used to implement lie‐detection (Davatzikos et al., 2005). A variety of unsupervised methods including PCA, K‐means clustering, ICA, etc. have also been used for exploratory analysis of fMRI data (Beckmann, DeLuca, Devlin, & Smith, 2005; Calhoun, Maciejewski, Pearlson, & Kiehl, 2008; Goutte, Toft, Rostrup, Nielsen, & Hansen, 1999; Hansen et al., 1999; Mezer, Yovel, Pasternak, Gorfine, & Assaf, 2009).

Support vector machines are a powerful set of machine‐learning methods that can be used to analyze data and recognize patterns (Cristianini and Shawe‐Taylor, 2000; Hamel, 2009; Vapnik, 2000). The SVM algorithm seeks a maximum margin separating hyperplane, thus making it resilient to overfitting. This means that they provide better generalization, allowing the best prediction accuracy for previously unseen test data. Also, when a linear kernel is used, they allow the possibility of generating discrimination maps so that they can be visualized in the original data space. That is, in fMRI analysis, the SVM weights can be superimposed back onto the original brain space so that the most significant weights can be visually traced back to the most discriminatory parts of the brain. Thus, SVMs help not only in effective pattern discrimination but also in pattern localization in the given data, hence improving interpretability of the model. SVMs are a preferred method when the data are very high dimensional, that is, it has many more features than the number of examples, which is typically the case for fMRI experiments, where each voxel represents a different feature. SVM explores this high‐dimensional space for an optimal separating hyperplane and the examples that are closest to the hyperplane are called the support vectors. SVMs have been shown to be effective for identifying brain states in previous studies (LaConte et al., 2005; Mitchell et al., 2004).

The objective of this work is to compare and contrast AVAST with the other more popular image acquisition techniques, BOLD and perfusion‐weighted ASL, in the setting of a predictive machine‐learning framework. Owing to the advantageous characteristics noted above, we choose SVM classification as our machine‐learning technique to compare AVAST performance to BOLD and perfusion‐weighted ASL in terms of SVM classification accuracy. To that end, we employed the SVM algorithm to perform temporal brain‐state classification of motor‐visual activation versus rest using images captured by each of the three acquisition techniques: BOLD, ASL, and AVAST. We have presented their comparative performance in terms of classification accuracy and significant model weights.

## 2 Methods

### 2.1 Stimulation paradigm

Ten healthy subjects participated in this study after signing a written consent and were scanned in accordance with the local IRB. The subjects included six men and four women between the ages of 20 and 35 years. They were given mirrored glasses to view a rear projection screen while being scanned. The paradigm involved displaying five cycles of alternating 30‐s blocks of flashing checkerboard (8 Hz) and static fixation cross (total duration = 300 s). The subjects performed a robust visuospatial activation task by doing self‐paced finger tapping with their right hand when presented with the flashing checkerboard and rest when presented with the fixation cross. The experiment was performed twice per subject and two runs of data were acquired using each acquisition technique.

### 2.2 Data acquisition

All functional images were collected on a 3T GE Signa Excite scanner. Images were captured using each of the three acquisition techniques (BOLD, ASL, AVAST) on every subject while they performed the activation task. To ensure that the steady state was reached, four dummy scans were collected at the start of each run. Image acquisition details were as follows:

BOLD: A single‐shot gradient echo reverse spiral pulse sequence was used (TR/TE/FA/FOV = 2 s/30 ms/90°/24 cm, 64 × 64 matrix, 11 contiguous slices to match ASL and AVAST).
Perfusion‐weighted ASL: Images were acquired using a functional CBF scheme employing an off‐resonance corrected PCASL technique (Jahanian, Noll, & Hernandez‐Garcia, 2011) followed by a 3D stack of spirals acquisition (Nielsen & Hernandez‐Garcia, 2012) (TR/TE/FOV = 4 s/3 ms/24 cm, tagging duration = 2 s, postinversion delay = 1.5 s, 64 × 64 matrix, 11 contiguous slices, slice thickness = 6 mm, bandwidth = 125 kHz, duration of 3D spiral readout = 385 ms).
AVAST: The same pulse sequence utilized for the perfusion‐weighted scans was used here, but the tagging parameters were modified to achieve arterial blood weighting. First, a calibration scan was implemented to find the optimal timing parameters (tagging duration and TR) tailored for each subject as in Jahanian et al. (2015). This optimization process was automated, and the parameters found were then used to acquire images using the functional aCBV scheme of AVAST (i.e., adjust TR and tagging duration obtained from the calibration scan, no postinversion delay).

### 2.3 Preprocessing steps

All datasets were reconstructed and the following preprocessing steps were performed before analysis using the SVM training and testing setup.

BOLD: A custom MATLAB code was used for k‐space spike removal and spiral reconstruction. SPM8 (http://www.fil.ion.ucl.ac.uk/spm, RRID: SCR_007037) was used to perform the following: (1) slice timing correction, which corrects for differences in acquisition time between slices during sequential imaging; (2) rigid body motion correction; and (3) spatial smoothing using a Gaussian smoothing kernel with FWHM of 8 mm.

Arterial spin labeling and AVAST: As before, custom MATLAB code was used for k‐space spike removal and 3D spiral reconstruction and SPM8 motion correction was performed. Next, the resulting time series of images were surround subtracted and analyzed by estimation of standard GLM using the custom‐written software FASL01 (http://fmri.research.umich.edu/resources/software/shared_code.php). Spatial smoothing was then done using a Gaussian smoothing kernel with FWHM of 8 mm.

All analyses were performed in native space. Furthermore, the time course of each voxel was normalized by subtracting its mean over time and dividing by its standard deviation.

### 2.4 Features and examples

A classifier function outputs a binary class label for every input set of feature values. The features represent an example, whereas the label signifies the class that a particular example belongs to. More specifically, if **x** is an example with features [*x*_1_, *x*_2_, *x*_3_,…] and the class label is denoted by *y* = (±1), then the classifier function *f*() computes the label for every given input, that is *y* = *f*(*x*).

In our study, at each time point, a brain activation volume is acquired (using one of the three acquisition techniques). Each such volume is used as a separate example in which the voxel gray scale intensities act as features. Depending on whether the subject was tapping their finger or resting, a label of +1 or −1 is associated with each example.

Data acquired during one of the two runs is used as training data, while the other separate run is used as testing data. In the training phase, a mapping is learned from the training examples to the respective class labels and a classifier is built. In the testing phase, this model is used to predict the class of a previously unseen example from the testing data. Classifier performance is calculated as the ratio of the number of correctly classified test examples to the total number of test examples. At first, run 1 was used for training the classification model, whereas run 2 was used to assess the effectiveness of the model and then vice versa.

### 2.5 Dataset dimensionality

Each of the acquired three‐dimensional volumes, which act as training and testing examples, was of size [64 × 64 × 11] voxels. Initially, this amounted to 45,056 features that were then reduced to ~8,000 features by excluding all voxels that lay outside the brain region by using a brain mask created for each individual subject. For BOLD images, this mask was computed by including only those voxels that were within one standard deviation of the mean of the mean image. For ASL and AVAST, all voxels within one standard deviation of the mean of the baseline image (i.e., the mean of the control images in the time series) were included. For the AVAST method, the timing parameter TR was tailored separately to suit each subject and a brief account of the optimal parameters is listed (Table 1). Note that, for each of the techniques, a different number of examples were collected due to the different TR in each technique (Table 2). The number of examples also varies because of the use of surround subtraction in ASL and AVAST. Two such runs were collected for each of the 10 subjects resulting in a total of 20 runs of data.

**Table 1:** Number of subjects corresponding to each tailored repetition time (TR) using arterial volume‐weighted arterial spin tagging

Tailored TR (s)	No. of subjects
2.0	2
2.1	2
2.2	2
2.4	3
2.5	1

**Table 2:** Number of examples in train and test datasets corresponding to each repetition time (TR) using each acquisition method

Technique	TR (s)	No. of examples
BOLD	2	150
ASL	4	74
AVAST	1.9	156
AVAST	2.0	148
AVAST	2.1	142
AVAST	2.2	134
AVAST	2.4	124
AVAST	2.5	118
sBOLD	4^a^	75
sAVAST	3.8–5^a^	59–78

sBOLD and sAVAST stand for subsampled runs of BOLD and AVAST.

AVAST, arterial volume‐weighted arterial spin tagging; ASL, arterial spin labeling; sBOLD, subsampled blood oxygenation–level dependent.

a Effective TR after subsampling.

Runs acquired with perfusion‐weighted ASL technique (TR = 4 s) included fewer time points than those acquired using BOLD (TR = 2 s) or AVAST (TR = 1.9–2.5 s). In order to address this discrepancy, the analysis was repeated for subsampled runs of BOLD and AVAST such that only every other time point was considered during analysis. These are denoted as subsampled BOLD (sBOLD) and sAVAST, respectively. sBOLD run had an effective TR of 4 s, whereas the sAVAST runs had an effective TR that ranged from 3.8 to 5 s depending on the optimal TR for that subject.

### 2.6 SVM classification

In standard SVM classification approach, a separating boundary between the two classes of examples (e.g., +1 and −1) is learned such that the distance (termed “margin”) between the data points and boundary is maximal. In higher dimensions, the separating boundary manifests itself as a hyperplane. This separating hyperplane generated by the SVM algorithm is orthogonal to the weight vector **w** which defines the direction in which the examples of the two classes differ most from one another. Thus, the classifier is parameterized by **w**, which can be solved for by using the following optimization problem:

$$\min_{w}^{}\left({\left\|w\right\|}^{2}+C\sum_{i=1}^{n}\xi_{i}\right),$$

(1) s.t.∀i∈{1,2,…,n}:

$$y_{i}w^{T}x_{i}\geq 1-\xi_{i}\;\&\;\xi_{i}\geq 0,$$

where **w** is the normal vector to the hyperplane, **y**_*i*_ are the known input class labels, **x**_*i*_ are the input feature vectors, *C* is the trade‐off parameter used to penalize misclassifications, and ξ_*i*_ are the nonnegative slack variables which measure the degree of misclassification of the input data **x**_*i*_. The SVM then uses the sign of the decision function *f*(*x*) = **w** ^T^ **x** to classify any new data point x represented by the feature vector x into one class or the other.

LIBSVM (Chang & Lin, 2011, RRID:SCR_010243), a Library for Support Vector Machines, was used to perform the SVM classification with the default linear kernel and default value of *C* = 1.

### 2.7 Transition periods

**Figure 1:**
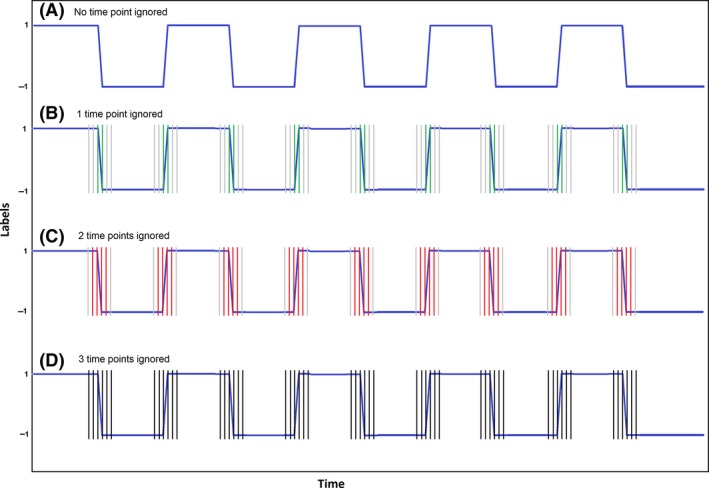
Schematic diagram depicting the transition points that are ignored. (A) No time points ignored, (B) one time point ignored—green, (C) two time points ignored—red, (D) three time points ignored—black

The paradigm design includes 10 alternating blocks of flashing checkerboard and fixation cross. Thus, there are nine intervals when the subject switches from one state to another (finger tapping or rest). These are called transition periods during which the vascular response to neuronal activation is still “ramping up” to its stable state in our block design experiment. Omitting the transition periods for both training and testing runs is common practice for offline studies (LaConte, Peltier, & Hu, 2007; LaConte et al., 2003). The exclusion of transition periods can improve the accuracy for the nontransition states. We investigated the effect of the transition period by excluding scans acquired during these periods from the modeling exercise as follows. Initially, the training was implemented using all the time points. Then, this exercise was repeated by excluding 1 time point from both blocks (last time point from previous block and first time point from next block) at each transition point. The same was further repeated by excluding two and three time points, respectively. This timing is illustrated in the Fig. 1.

### 2.8 Permutation tests

The weight vector **w** generated by the SVM algorithm is representative of the most discriminatory regions of the brain. When mapped back into original image space, this vector generates the discriminating volume also called the weight vector map. The weight vector map is, thus, a representation of the voxels that are most vital to the classification. The magnitude of the absolute value of each voxel weight determines its importance in discriminating the brain states, and the most important voxels for discrimination of the brain states can be inspected by merely thresholding the obtained weight vector map. A permutation test was employed to assess the reproducibility of these spatial patterns.

Briefly, permutation tests are nonparametric techniques that estimate the distribution of a statistic under a null hypothesis empirically and have been used with fMRI data previously (Jahanian, Hossein‐Zadeh, Soltanian‐Zadeh, & Ardekani, 2004; Mourão‐Miranda, Bokde, Born, Hampel, & Stetter, 2005; Nichols & Holmes, 2001). The null hypothesis proposes that there are no differences between the two brain states, and thus, the labels assigned to each example are inconsequential. The alternate hypothesis, on the other hand, claims that the assigned class labels are actually indicative of the brain state that an example belongs to and better than random. One can estimate the distribution of weights assigned to each voxel under the null hypothesis by randomly permuting the class labels multiple times and training the SVM each time with this different permutation of labels. In each instance, the weights were normalized to have unit standard deviation. The SVM training was also done once with the known correct nonpermuted labels. Now, for each voxel, the *p* value under null hypothesis was calculated as the ratio of number of times that the voxel weight assigned to it was greater than or equal to the weight assigned to it when training with original nonpermuted labels. Since we permuted the labels and trained 2,000 different models, if this number is smaller than 20, then that voxel is likely to be predictive of the class label with a significance level of 1%. The weight vector maps shown in the RESULTS display all significant voxels with *p* value <.01.

## 3 Results

### 3.1 Classification accuracy

**Figure 2:**
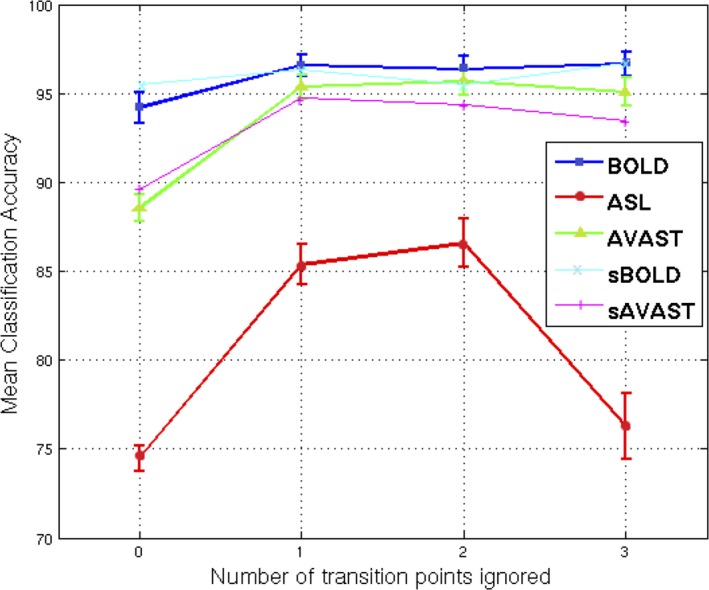
Mean classification accuracy over two runs of all 10 subjects for each acquisition technique (BOLD: blue, ASL: red, AVAST: green) against number of ignored transition points. The error bars depict standard error. BOLD, blood oxygenation–level dependent; AVAST, arterial volume‐weighted arterial spin tagging; ASL, arterial spin labeling

Figure 2 shows a plot of the mean classification accuracy across both runs of all 10 subjects. It demonstrates that the mean classification accuracy obtained by AVAST was consistently better than that offered by ASL and almost equivalent to BOLD. Ignoring transition points improves the classification accuracy initially but plateaus for BOLD and AVAST, whereas it deteriorated for ASL when three time points are ignored in each block. In order to ensure that the classifier power was not being driven by the number of examples in each case, sBOLD and subsampled AVAST (sAVAST) runs were also analyzed using the same setup. The mean classification accuracy for these analyses was found to be similar to the earlier case, as seen in the plot.

### 3.2 Weight vector maps

The SVM algorithm generated discriminatory weight maps and the permutation tests allowed us to find the significant voxels from these maps as described in the METHODS. These maps were indicative of the detection sensitivity of the acquisition technique.

**Figure 3:**
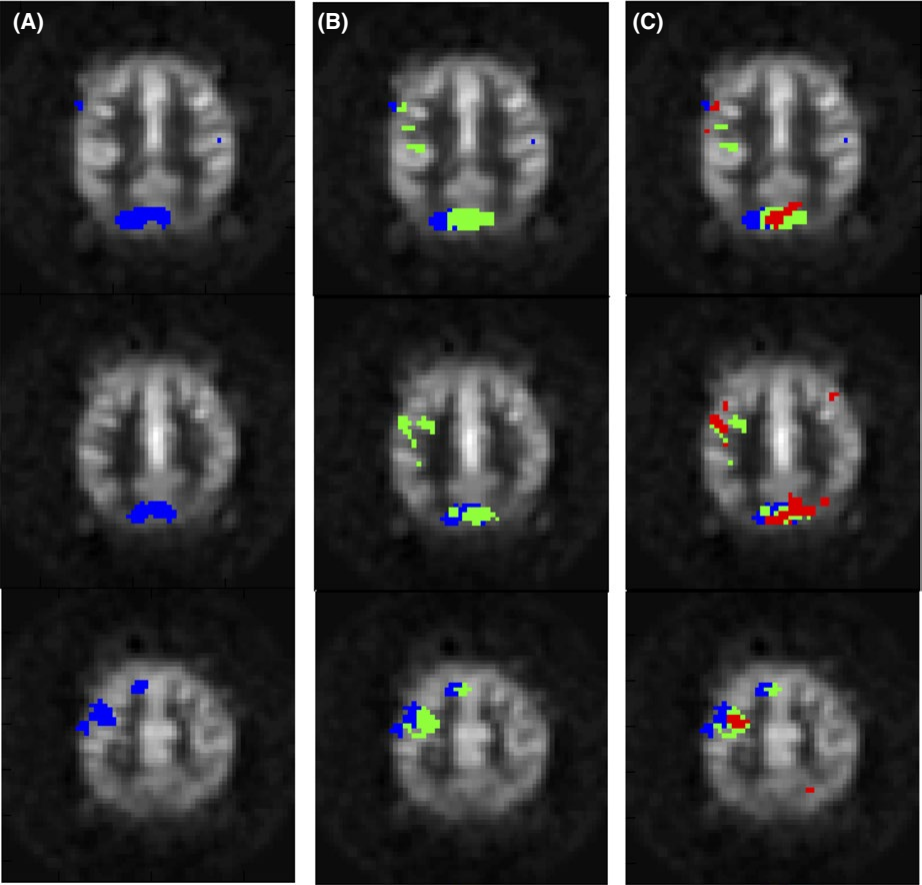
Significant support vector machine weights after permutation tests with *p* < .01 for a representative subject for each acquisition technique superimposed on a mean subtraction image as underlay (BOLD: blue, ASL: red, AVAST; green) (A) BOLD, (B) BOLD + AVAST, (C) BOLD + AVAST + ASL. BOLD, blood oxygenation–level dependent; AVAST, arterial volume‐weighted arterial spin tagging; ASL, arterial spin labeling

Figure 3 depicts select slices for a representative subject showing the most significant SVM weights with *p* < .01 in the left motor and premotor cortices as well as the visual cortex, as expected. The left‐most column shows SVM weights for BOLD (blue) technique. The middle column shows the AVAST (green) weights superimposed on BOLD and the right‐most column shows ASL (red) weights superimposed on BOLD and AVAST. The clusters of significant weights were bigger and more robust in the AVAST technique as compared to ASL.

## 4 Discussion

Traditionally, perfusion‐weighted ASL techniques suffer from low SNR and detection sensitivity. By taking advantage of the kinetics of the tag through the vasculature, AVAST facilitates the tailoring of the timing parameters for each subject. This permits the acquisition rate to be much faster and allows much superior temporal resolution as compared to standard perfusion‐weighted ASL. Thus, we can acquire a larger number of volumes in the same duration. Also, as noted previously in Jahanian et al. (2015), AVAST offers much better activation detection sensitivity. Both these features are advantageous for the machine‐learning techniques since it increases the degrees of freedom and also the images obtained are much more sensitive to activation. Thus, AVAST images exhibit better classification performance in terms of higher classification accuracy and more robust clusters of significant weights in the expected brain regions.

The data presented here indicate that AVAST images can be used for SVM classification more reliably than perfusion‐weighted ASL and are comparable to BOLD images in terms of their reliability. AVAST images, however, retain some of the advantages of ASL imaging, such as its robustness to scanner drifts and ability to be quantified. ASL images do not depend on T2* contrast, so they can use shorter TEs and thus mitigate susceptibility artifacts.

This study presents promising results that promote the use of machine‐learning techniques for brain‐state classification of images acquired by using the AVAST technique. This technique might be used for dynamic fMRI experiments and real‐time brain‐state classification studies as in Hernandez‐Garcia, Jahanian, Greenwald, Zubieta, & Peltier (2011).

## Conflict of Interest

None declared.
